# Safety of the human papillomavirus (HPV)-16/18 AS04-adjuvanted vaccine in adolescents aged 12–15 years: Interim analysis of a large community-randomized controlled trial

**DOI:** 10.1080/21645515.2016.1183847

**Published:** 2016-11-14

**Authors:** Matti Lehtinen, Tiina Eriksson, Dan Apter, Mari Hokkanen, Kari Natunen, Jorma Paavonen, Eero Pukkala, Maria-Genalin Angelo, Julia Zima, Marie-Pierre David, Sanjoy Datta, Dan Bi, Frank Struyf, Gary Dubin

**Affiliations:** a University of Tampere, Tampere, Finland; b Family Federation of Finland, Helsinki, Finland; c University of Helsinki, Helsinki, Finland; d GSK Vaccines, Wavre, Belgium; e GSK Vaccines, King of Prussia, PA, USA

**Keywords:** Human papillomavirus (HPV), HPV-16/18 AS04-adjuvanted vaccine, adolescents, safety, autoimmune disease, insulin-dependent diabetes mellitus

## Abstract

This community-randomized controlled trial was initiated to assess the overall and herd effects of 2 different human papillomavirus (HPV) immunization strategies in over 80,000 girls and boys aged 12–15 y in 33 communities in Finland (ClinicalTrials.gov NCT00534638). Overall, 14,838 adolescents received HPV-16/18 vaccine (2,440 boys and 12,398 girls) and 17,338 received hepatitis-B virus (HBV) vaccine (9,221 boys and 8,117 girls). In an interim analysis, vaccine safety was assessed by active monitoring and surveillance via health registry linkage. Active monitoring showed that the HPV-16/18 vaccine has acceptable safety and reactogenicity in boys. In all study participants, the observed incidences (per 100,000 person-years) of serious adverse events (SAEs) possibly related to vaccination were 54.3 (95% Confidence Interval [CI]: 34.0–82.1) in the HPV-16/18 group and 64.0 (95% CI: 43.2–91.3) in the HBV group. During the follow-up period for this interim analysis, the most common new-onset autoimmune diseases (NOADs; with incidence rate ≥15 per 100,000) in any group based on hospital discharge registry (HILMO) download were ulcerative colitis, juvenile arthritis, celiac disease, insulin-dependent diabetes mellitus (IDDM) and Crohn's disease. No increased NOAD incidences were observed in HPV-16/18 vaccine recipients compared to HBV vaccine recipients. In both the SAE possibly related- and HILMO-analyses, a lower incidence of IDDM was observed in HPV-16/18 vaccinees compared to HBV vaccinees (relative risks, 0.26 [95% CI: 0.03–1.24] and 0.16 [95% CI: 0.03–0.55], respectively).

## Introduction

Virus-like particle (VLP)-based vaccines against oncogenic, high-risk human papillomavirus (HPV) types 16 and 18 have been licensed in most countries, most recently including a 9-valent HPV vaccine (*Gardasil 9* [Merck & Co. Inc.]).^1-6^ Basis for the licensure of *Cervarix*® (GSK Vaccines) and *Gardasil* (Merck & Co., Inc.) was their acceptable safety profiles and vaccine efficacy (VE) against HPV types 16/18- or 6/11/16/18-related persistent genital infections and associated cervical intraepithelial neoplasia grade 2/3 (CIN2/3).^7,8^ Phase III trial VE estimates for the 2 vaccines appear similar against HPV types 16 and 18, but differ against CIN3+ irrespective of HPV type.^9^ While follow-up trials involving 22,000 young women (aged 16–19 y at the time of first vaccination) are evaluating the long-term safety and efficacy of the 2 vaccines,^10,11^ recently launched national vaccination programs will provide data on rare safety outcomes albeit gradually.^12-15^

The HPV-16/18 vaccine is formulated with the AS04 Adjuvant System, containing 3-*O*-desacyl-4′-monophosphoryl lipid A (MPL; 50 µg) adsorbed on aluminum salt (500 µg Al^3+^), which has been shown to widely induce T-helper and memory B cell responses,^16^ and probably is partially responsible for the wide cross-protectivity of the vaccine.^17,18^ However, a theoretical adverse event of this enhanced immunogenicity might be the induction (or promotion) of (ongoing) autoimmune processes. The overall and specific, with regard to new-onset autoimmune diseases (NOADs), safety profile of the HPV-16/18 AS04-adjuvanted vaccine has been acceptable in all ages studied and in combination with other routine vaccines.^19-24^ However, to date, safety data have been collected via conventional reporting of adverse events (AEs) and serious adverse events (SAEs) in clinical trials, and quantitative safety data from large cohorts of HPV-16/18 vaccinated adolescents and population-based health registers are sparse.

We report interim safety results for the HPV-16/18 AS04-adjuvanted vaccine administered to a large cohort of adolescents (ClinicalTrials.gov NCT00534638), with special emphasis on unique, quantitative population-based health care registry data on occurrence of NOADs post-vaccination. Study design and population characteristics have been described in a previous publication.^25^ The primary objective of the study is to demonstrate the effectiveness of the HPV-16/18 vaccine in reducing the prevalence of HPV-16/18 genital infection in females following community-based vaccination of girls and boys 12–15 y old; these results will be reported in a future publication upon study and analysis completion.

## Results

### Study population

**Figure 1.**
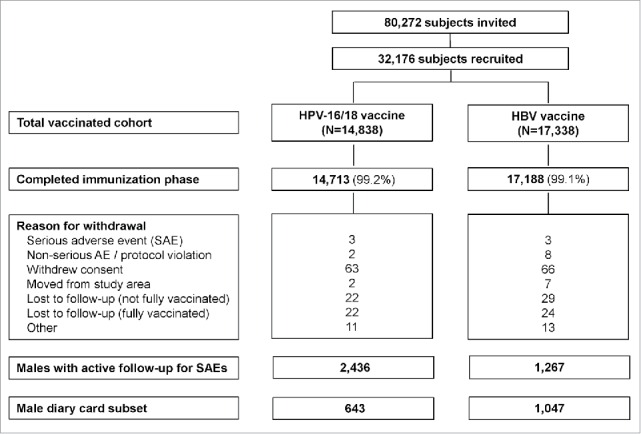
Subject disposition. N, number of subjects; SAE, serious adverse event; AE, adverse event.

A total of 80,272 adolescents (40,852 boys and 39,420 girls) in the 1992–1995 birth cohorts were invited to participate in the study between October 2007 and April 2010, of whom 32,176 (11,661 boys and 20,515 girls) were enrolled and received at least one dose of study vaccine (Fig. 1). In all, 14,838 adolescents received the HPV-16/18 vaccine (2,440 boys and 12,398 girls) and 17,338 received the HBV vaccine (9,221 boys and 8,117 girls) at 12–16 y of age. Virtually all subjects were white (Caucasian/European heritage; 99.0%) (Table S1).

### Active safety surveillance in males

**Figure 2.**
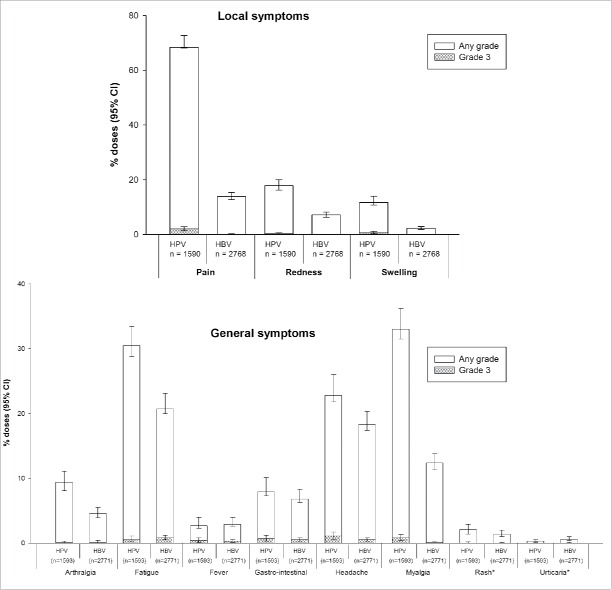
Incidence of solicited local and general symptoms reported during the 7-day post-vaccination period (Days 0–6) following any vaccine dose (male study participants in the male diary card subset, total vaccinated cohort). *Occurrence of rash and urticaria within 30 minutes following vaccination.

The male diary card subset comprised 643 subjects who received HPV-16/18 vaccine and 1,047 who received HBV vaccine (Fig. 1). Compliance was high in returning general and local symptoms sheets (83.5% and 83.3% overall per dose, respectively, in the HPV-16/18 group and 88.6% and 88.5%, respectively, in the HBV group). Pain at the injection site was the most frequently solicited local symptom in both groups (reported during the 7-day period following 70.4% and 14.0% of HPV-16/18 and HBV vaccine doses, respectively); fatigue (31.1% and 21.5%), headache (23.9% and 18.8%) and myalgia (33.8% and 12.5%) were the most frequently solicited general symptoms. The rate of local and some general solicited symptoms (arthralgia, fatigue, headache and myalgia) appeared higher among boys who received HPV-16/18 vaccine compared to those who received HBV vaccine (Fig. 2).

Unsolicited AEs reported in the month following any vaccine dose were comparable between HPV-16/18- and HBV-vaccinated boys (Table 1). During the 30-day post-vaccination period, 24.4% and 19.3% of boys reported at least one unsolicited symptom after 10.1% and 7.6% of doses in the HPV-16/18 and HBV groups, respectively. Unsolicited AEs considered by the investigator to be possibly related to vaccination were reported by 1.9% and 1.8% of boys in the HPV-16/18 and HBV groups, respectively, after 0.7% and 0.6% of doses.

**Table 1.** Active safety surveillance from Month 0–Month 12 (male dairy card subset, total vaccinated cohort). Incidence of unsolicited symptoms (most frequent symptoms listed), serious adverse events, medically significant conditions and new-onset autoimmune diseases.

	HPV-16/18 vaccine (N = 643)		HBV vaccine (N = 1047)	
	n	% (95% CI)	n	% (95% CI)
***Within 30 d (Days 0–29) following any vaccine dose***				
Unsolicited symptoms	157	24.4 (21.1–27.9)	202	19.3 (16.9–21.8)
Nasopharyngitis	33	5.1 (3.6–7.1)	17	1.6 (0.9–2.6)
Headache	23	3.6 (2.3–5.3)	16	1.5 (0.9–2.5)
Oropharyngeal pain	15	2.3 (1.3–3.8)	32	3.1 (2.1–4.3)
Pyrexia	17	2.6 (1.5–4.2)	21	2.0 (1.2–3.0)
Upper RTI	13	2.0 (1.1–3.4)	3	0.3 (0.1–0.8)
Vaccine-related	12	1.9 (1.0–3.2)	19	1.8 (1.1–2.8)
***Up to Month 12 after first vaccine dose***				
Serious adverse events	8	1.2 (0.5–2.4)	21	2.0 (1.2–3.0)
Vaccine-related	1	0.2 (0.0–0.9)	1	0.1 (0.0–0.5)
Medically significant conditions	47	7.3 (5.4–9.6)	76	7.3 (5.8–9.0)
New onset autoimmune diseases	1	0.2 (0.0–0.9)	1	0.1 (0.0–0.5)

N, total number of male subjects; n (%), number (percentage) of male subjects with at least one symptom; HPV vaccine, HPV-16/18 AS04-adjuvanted vaccine; HBV, hepatitis B virus vaccine; RTI, respiratory tract infection.

From Dose 1 to Month 12, in male study participants in the diary card subset, the proportion of boys with SAEs, SAEs possibly related to vaccination, NOAD and medically significant conditions (MSCs) were within the same range in both vaccine groups (Table 1). SAEs were reported for 1.2% and 2.0% of boys who received HPV-16/18 and HBV vaccines, respectively. Two SAEs (IDDM and juvenile arthritis [JA]) were considered possibly related to vaccination by the investigator (blinded to treatment allocation). MSCs were reported by 7.3% of boys for both vaccines. These were considered possibly related to vaccination by the investigator in 3 boys (0.5%) who received HPV-16/18 vaccine and 1 boy (0.1%) who received HBV vaccine (concussion, IDDM, JA and oropharyngeal pain; blinded to treatment allocation). One boy in each group reported an NOAD (IDDM and JA). Both events were reported as SAEs and were considered possibly related to vaccination by the investigator.

From Dose 1 to Month 12, in male study participants with active follow-up, SAEs were reported by 2.4% of boys who received HPV-16/18 vaccine and 2.0% of boys who received HBV vaccine. Possible relationship of SAEs with vaccination could not be excluded in 4 boys (0.2%) who received HPV-16/18 vaccine and 1 boy (0.1%) who received HBV vaccine (abdominal pain, ulcerative colitis, IDDM and JA; blinded to treatment allocation).

### SAEs considered possibly related to vaccination in all study participants based on active and passive surveillance

During the follow-up period for this interim analysis, the observed incidence rates (per 100,000 person-years) for SAEs considered possibly related to vaccination according to the investigator in all study participants based on active and passive surveillance were 54.3 (95% confidence interval [CI]: 34.0–82.1) in the HPV-16/18 group and 64.0 (95% CI: 43.2–91.3) in the HBV group. The most common SAEs considered possibly related to vaccination by the investigator (with incidence rate ≥10 per 100,000 person-years in any group) were ulcerative colitis, Crohn's disease, and IDDM. No deaths had been reported at the time of this interim analysis.

The incidence rates of possibly vaccine-related SAEs were 52.8 (95% CI: 31.3–83.4) and 72.3 (95% CI: 41.3–117.3) in the HPV-16/18 and HBV groups, respectively, for female study participants (based on spontaneous reporting and Care Register for Social Welfare and Health Care [HILMO] data) and 62.1 (95% CI: 16.9–158.9) and 56.6 (95% CI: 30.9–94.9), respectively, for male study participants (based on active surveillance in a subset of subjects as well as spontaneous reporting and HILMO data for all subjects).

### New-onset autoimmune diseases in all study participants

**Figure 3.**
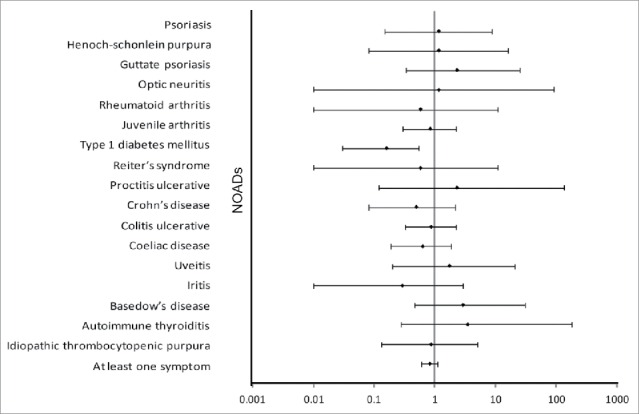
Estimated relative risk of the occurrence of new-onset autoimmune disease (NOADs) with 95% confidence interval, classified by MedDRA primary system organ class and preferred term, during the entire study period (all study participants, total vaccinated cohort). Dots represent point estimates. NOADS which occur in only one group remain blinded and are therefore not shown in this figure.

During the follow-up period for this interim analysis in all study participants, the most common NOADs (with incidence rate ≥15 per 100,000 in any group based on the HILMO linkage) were ulcerative colitis (25.0 and 29.0 cases per 100,000 person years in the HPV-16/18 and HBV groups, respectively), juvenile arthritis (22.2 and 26.6 cases per 100,000 person years, respectively), celiac disease (16.7 and 26.6 cases per 100,000 person years, respectively), IDDM (8.3 and 50.7 cases per 100,000 person years, respectively), and Crohn's disease(8.3 and 16.9 cases per 100,000 person years, respectively). Of note, no cases of Guillain-Barré syndrome were observed, and other NOADs were found only occasionally in both vaccine groups. In terms of numbers of cases, IDDM was the most common NOAD diagnosis identified in the HILMO linkage (3 cases in the HPV-16/18 group and 21 in the HBV group). Almost all the 95% CIs of relative risk (RR) estimates for the incident NOAD cases in the HPV-16/18 group as compared to the HBV group included unity (Fig. 3), with the exception of IDDM (RR: 0.16, 95% CI: 0.03–0.55). A decreased relative risk of IDDM was observed both for females (2 vs. 7 cases; RR: 0.19, 95% CI: 0.02–0.97) and males (1 vs. 14 cases; RR: 0.27, 95% CI: 0.01–1.8) (Table S2).

The incidences of the most common NOADs identified by the HILMO linkage (i.e., medical history-confirmed) and by SAE reporting are shown in Table 2. More cases were recorded in the HILMO registry (Table 2).

**Table 2.** Incidence (per 100,000 person years) and relative risk of major NOADs in all study participants (total vaccinated cohort) based on active and passive surveillance and registry analysis.

		HPV-16/18 vaccine All		HBV vaccine All		Relative risk (HPV/HBV) (95% CI)		
Category	Case identification	No. cases	Incidence (10^5^) (95% CI)	No. cases	Incidence (10^5^) (95% CI)	All	Female^d^	Male^d^
Henoch-Schonlein purpura	Registry all^a^	2	5.6 (0.7–20.1)	2	4.8 (0.6–17.5)	1.15 (0.08–15.87)	1.3 (0.07–76.47)	na
	Registry 12 m^b^	2	9.3 (1.1–33.7)	1	4.0 (0.1–22.2)	2.34 (0.12–138.23)	1.31 (0.07–77.42)	na
	SAE possibly-related^c^	1	2.5 (0.1–13.7)	1	2.1 (0.1–11.9)	1.16 (0.01–90.77)	0.65 (0.01–50.96)	na
Idiopathic thrombocytopenic purpura	Registry all^a^	3	8.3 (1.7–24.4)	4	9.7 (2.6–24.7)	0.86 (0.13–5.10)	0.97 (0.11–11.64)	na
	Registry 12 m^b^	1	4.7 (0.1–26.0)	2	8.0 (1.0–28.8)	0.59 (0.01–11.25)	0.33 (0.01–6.3)	na
	SAE possibly-related^c^	1	2.5 (0.1–13.7)	1	2.1 (0.1–11.9)	1.16 (0.01–90.77)	0.65 (0.01–50.96)	na
Autoimmune thyroiditis	Registry all^a^	3	8.3 (1.7–24.4)	1	2.4 (0.1–13.5)	3.45 (0.28–181.16)	1.94 (0.16–102.07)	na
	Registry 12 m^b^	3	14.0 (2.9–40.9)	1	4.0 (0.1–22.2)	3.51 (0.28–184.51)	1.97 (0.16–103.34)	na
	SAE possibly-related^c^	*1*	na	*1*	na	na	na	na
Celiac disease	Registry all^a^	6	16.7 (6.1–36.3)	11	26.6 (13.3–47.5)	0.63 (0.19–1.85)	0.56 (0.15–1.93)	na
	Registry 12 m^b^	3	14.0 (2.9–40.9)	8	31.9 (13.8–62.8)	0.44 (0.08–1.83)	0.39 (0.06–2.02)	na
	SAE possibly-related^c^	—	na	—	na	na	na	na
Ulcerative colitis	Registry all^a^	9	25.0 (11.4–47.5)	12	29.0 (15.0–50.6)	0.86 (0.32–2.23)	1.13 (0.29–5.28)	0.96 (0.1–4.8)
	Registry 12 m^b^	6	28.0 (10.3–61.0)	9	35.9 (16.4–68.1)	0.78 (0.23–2.46)	0.82 (0.18–4.13)	0.77 (0.02–6.86)
	SAE possibly-related^c^	5	12.3 (4.0–28.8)	3	6.4 (1.3–18.7)	1.93 (0.37–12.41)	1.3 (0.19–14.35)	3.84 (0.05–301.44)
Crohn's disease	Registry all^a^	3	8.3 (1.7–24.4)	7	16.9 (6.8–34.8)	0.49 (0.08–2.16)	1.94 (0.16–102.07)	na
	Registry 12 m^b^	2	9.3 (1.1–33.7)	5	19.9 (6.5–46.5)	0.47 (0.04–2.86)	1.31 (0.07–77.42)	na
	SAE possibly-related^c^	1	2.5 (0.1–13.7)	5	10.7 (3.5–24.9)	0.23 (0.00–2.07)	0.65 (0.01–50.96)	na
IDDM	Registry all^a^	3	8.3 (1.7–24.4)	21	50.7 (31.4–77.5)	0.16 (0.03–0.55)	0.19 (0.02–0.97)	0.27 (0.01–1.8)
	Registry 12 m^b^	3	14.0 (2.9–40.9)	8	31.9 (13.8–62.8)	0.44 (0.08–1.83)	0.26 (0.02–1.6)	1.28 (0.02–15.93)
	SAE possibly-related^c^	2	4.9 (0.6–17.8)	9	19.2 (8.8–36.4)	0.26 (0.03–1.24)	0.16 (0–1.64)	0.77 (0.02–6.86)
Juvenile arthritis	Registry all^a^	8	22.2 (9.6–43.8)	11	26.6 (13.3–47.5)	0.84 (0.29–2.28)	0.49 (0.14–1.6)	2.56 (0.21–22.31)
	Registry 12 m^b^	7	32.7 (13.1–67.3)	11	43.8 (21.9–78.4)	0.75 (0.25–2.11)	0.41 (0.11–1.42)	2.56 (0.21–22.33)
	SAE possibly-related^c^	*3*	na	*3*	na	na	na	na
Rheumatoid arthritis	Registry all^a^	1	2.8 (0.1–15.5)	2	4.8 (0.6–17.5)	0.58 (0.01–11.05)	0.32 (0.01–6.22)	na
	Registry 12 m^b^	1	4.7 (0.1–26.0)	1	4.0 (0.1–22.2)	1.17 (0.01–91.96)	0.66 (0.01–51.51)	na
	SAE possibly-related^c^	*1*	na	*1*	na	na	na	na

a health register-identified, medical record-confirmed cases diagnosed post 1^st^ vaccine dose during the entire study period (all study participants, total vaccinated cohort); 35,992 and 41,401 follow-up years in the HPV-16/18 and HBV groups, respectively

b health register-identified, medical record-confirmed cases diagnosed within 12 months after last vaccination (all study participants, total vaccinated cohort); 21,422 and 25,097 follow-up years in the HPV-16/18 and HBV groups, respectively

c SAE cases considered possibly related to vaccination during the entire study period (all study participants, total vaccinated cohort); 40,552 and 46,890 follow-up years in the HPV-16/18 and HBV groups, respectively.

*n*, total number of cases reported, information is blinded since the study is ongoing

HPV-16/18 vaccine, HPV-16/18 AS04-adjuvanted vaccine; HBV, hepatitis B virus vaccine; IDDM, insulin-dependent diabetes mellitus ; na, not available; 12 m, 12 months; 95% CI, 95% confidence interval

### Pregnancies in all female study participants

A total of 174 pregnancies had been spontaneously reported at the time of this interim analysis (100 in girls who received HPV-16/18 vaccine and 74 in girls who received HBV vaccine). The majority of pregnancies resulted in elective termination with no apparent congenital anomaly (60 [60.0%; 95% CI: 49.7–69.7] and 52 [70.3%; 95% CI: 58.5–80.3] for the 2 vaccines, respectively) or in a live infant (no premature birth) with no apparent congenital anomaly (30 [30.0%; 95% CI: 21.2–40.0] and 20 [27.0%; 95% CI: 17.4–38.6], respectively). Spontaneous abortions before 22 weeks of gestation (with no apparent congenital anomaly) were reported in 7 girls who received HPV-16/18 vaccine versus 2 girls who received HBV vaccine (7.0% [95% CI: 2.9–13.9] vs. 2.7% [95% CI: 0.3–9.4]; RR: 2.59 [95% CI: 0.55–12.11]). No stillbirths, therapeutic abortions, ectopic pregnancies and molar pregnancies were reported. At the time of the reported analysis, 2 pregnancies were still ongoing in girls who received HPV-16/18 vaccine.

Complete pregnancy data from medical birth registries will be available at the time of final analysis.

## Discussion

Sizeable cluster-randomized phase IV trial cohorts, enrolled in a population-based fashion, are now available for studies on the effectiveness and safety of HPV-16/18 vaccination. These population-based cohorts provide unique health registry-based, quantitative data on the safety of the HPV-16/18 AS04-adjuvanted vaccine and the HBV vaccine. This large community-randomized study assessed the safety of HPV-16/18 vaccination in early adolescents, providing relative risk estimates on the occurrence of NOADs in the 2 vaccine arms over 2–3 y of follow-up. We found that the medical record-confirmed IDDM incidence in HPV-16/18 vaccinees was lower than that in HBV vaccinees. The incidence of IDDM seen in HPV-16/18 vaccinees in this study is also lower than that reported in somewhat younger, 10–14 year-old Finns (50.4 per 100,000).^26^ Our observation, and a significantly decreased incidence rate ratio for IDDM (0.55) in approximately 190,000 females that has also been reported following HPV-6/11/16/18 vaccination,^27^ suggest that the possible role of HPVs in IDDM warrants further investigation. The possibility, that the HPV VLP vaccines and/or the AS04-adjuvant of the HPV-16/18 vaccine provide unexpected protection against IDDM, e.g., by delaying/re-directing an already ongoing autoimmune process, should also be explored.

As for other NOADs, except those that remained blinded at the time of this analysis, we observed no apparent difference in the incidence of overall medical record-confirmed or possibly related occurrence estimates between the 2 vaccination arms. This is in line with comparable health registry-based findings on the HPV-6/11/16/18 vaccine.^28^ The health registry-based surveillance was more sensitive than the conventional SAE surveillance. This kind of health registry-based quantitative analysis is an important adjunct to the safety data on the HPV-16/18 vaccine and will in the future continue to be most appealing to health authorities in the context of newly licensed vaccines for suspected or alleged vaccine-associated adverse effects (e.g., autism, narcolepsy) or in the case of HPV vaccination, primary ovarian failure syndrome^29^ or miscarriage.^23,30-32^

Both active and passive safety surveillance in this trial showed that the HPV-16/18 vaccine has an acceptable safety and reactogenicity profile in boys, which is similar to the results of other studies of this vaccine in adolescent girls^24,33^ and boys.^34^

Strengths of our study were uniform enrolment of the early adolescents by school year and birth cohort and community, retaining the participants in the follow-up phase, and population-based health registry follow-up. Analysis of questionnaire data for the study participants showed no differences in behavioral or health conscience characteristics between the 2 vaccine groups.^25^ The differential blinding used in the study is a potential limitation, mainly for the active surveillance. Other limitations include the imbalance in gender distribution between the 2 vaccine groups (83.6% of vaccinees were female and 16.4% were male in the HPV-16/18 vaccine group compared with 46.8% and 53.2%, respectively, in the HBV vaccine group). In addition, safety data were collected via different methods of surveillance, including active surveillance in a subset of male study participants (using diary cards and phone calls) and surveillance of NOADs in all study participants based on download from the national heath registry (HILMO). The follow-up time for safety endpoints varied for the different surveillance methods and also within subjects for surveillance based on registry download. The HILMO database is one of the most important registers used in healthcare research in Finland.^35^ This database receives annually (by law) discharge information from all hospitals in the country, including both inpatient and outpatient services. However, it should be noted that the case assessment of HILMO-retrieved NOADs was based on the review of medical records which did not always provide all sufficient information for assessment of autoimmune diseases. Finally, analysis of safety was planned to be descriptive and no correction for multiplicity was applied. Study findings should therefore be interpreted with caution.

With the current follow-up mode, our community-randomized phase IV trial will continue to provide population-based safety data (e.g. on chronic diseases, pregnancy outcomes) for the HPV-16/18 vaccine.

## Materials and methods

### Study design

This study was conducted in 33 major, non-adjacent Finnish communities as previously described.^25^ All 80,272 Finnish or Swedish speaking boys and girls in the 1992–1995 birth cohorts in these communities were invited by letter to participate in the trial. The communities were stratified into 3 groups according to HPV-16/18 seroprevalence (low, <20.5%; intermediate, 20.5–24.0%; high, >24.0%). Within each seroprevalence group, communities were randomly assigned in equal numbers to one of 3 intervention arms using a random number generator. In Arm A communities, 90% of participating girls and boys received HPV-16/18 vaccine and 10% received hepatitis B virus (HBV) vaccine (*Engerix™ B*, GSK Vaccines); in Arm B communities, 90% of girls received HPV-16/18 vaccine and 10% of girls and 100% of boys received HBV vaccine; in Arm C communities, 100% of girls and boys received HBV vaccine. The 9:1 ratio used to allocate study participants from Arms A (males and females) and B (females only) was determined to target an HPV vaccination coverage of approximately 70% within adolescent resident in these communities, considering that 85–90% of study participants would meet admission criteria and that approximately 90% of them would agree to participate in the immunization phase. Treatment allocation at investigator sites was performed using a central internet-based randomization system. Vaccine doses were administered at Months 0, 1 and 6 at schools by the same study nurses during school years 2007–2008, 2008–2009 and 2009–2010. Although the study was open at community level because different vaccination strategies were used between Arms (for primary objectives), blinding was maintained for all subjects in Arm A and for girls in Arm B in order to reduce bias for both safety and effectiveness analysis.

The study was approved by a regional independent ethics committee and conducted in accordance with good clinical practice (GCP) and all applicable regulatory requirements, including the Declaration of Helsinki. Subjects aged <15 y provided written informed assent prior to the performance of any study-specific procedures; written informed consent was also obtained from participants' parents or legal representatives. Written informed consent was obtained from study participants aged ≥15 years.

### Safety reporting

**Figure 4.**
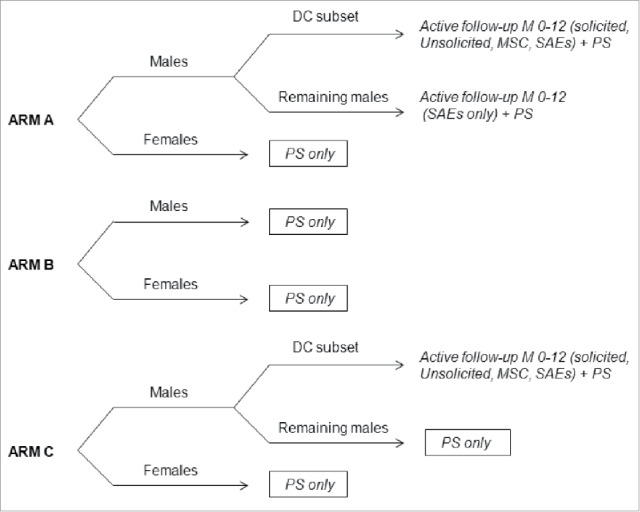
Overview of safety assessments within intervention arms. In Arm A, 90% of boys and girls received HPV-16/18 vaccine and 10% received HBV vaccine. In Arm B, 90% of girls received HPV-16/18 vaccine and 10% of girls and all boys received HBV vaccine. In Arm C, all girls and boys received HBV vaccine. DC, diary card; M, month; MSC, medically significant conditions; NOAD, new-onset autoimmune diseases; PS, passive surveillance including reporting of possibly vaccine-related SAEs and NOADs based on registry download; SAEs, serious adverse events.

A combination of active and passive safety surveillance was performed according to intervention arm (Fig. 4). As the study was conducted while the HPV-16/18 vaccine was not licensed for use in males, it was designed to provide important safety information in males in order to complement the already substantial safety data available in women. Therefore, a subset of boys from Arm A and Arm C communities was selected for active assessment of safety using diary cards (male diary card subset). The size of the eligible population was the main consideration for the selection of the communities for the diary card subset. These subjects were to record solicited local and general symptoms within 7 d (Days 0–6) and unsolicited AEs within 30 d (Days 0–29) after each vaccination. The investigator recorded any occurrence of rash and/or urticaria within 30 minutes following each vaccination. These subjects were also actively questioned at Months 7 and 12 about any MSCs (including NOADs) and SAEs that might have occurred after the first vaccination. Active safety surveillance for the occurrence of SAEs was also performed in boys from Arm A communities who were not included in the diary card subset at Months 7 and 12. A SAE was any untoward medical occurrence that resulted in death, was life-threatening, required hospitalization or prolongation of existing hospitalization, resulted in disability or incapacity, or was a congenital anomaly or birth defect in the offspring of a study participant.

Passive safety surveillance was performed for all subjects in all intervention arms throughout the study period up to the cut-off date for this interim analysis. Any SAEs reported to the investigator considered possibly related to vaccination and pregnancy outcomes were reported using a remote data entry system at the study sites and/or at the junior high-schools. For the HILMO-based analysis (Fig. S1), the study participants had consented for personal identity code (PIC)-based linkages of the Registry of Vaccinated Individuals (RVI) and the HILMO. The RVI and HILMO registry linkage for predefined NOADs (Table S3) was performed after the HILMO registry had been completed for the 2010 diagnostic (ICD-10 encoded) data on all outpatient and inpatient visits in Finland. Thus, depending on the time of enrolment, there were approximately 1–3 y of registry-based follow-up data available for the different birth cohorts for the predefined NOADs after administration of the first vaccine dose (from October 2007 to December 2010 for the 1992–1993 birth cohorts and from August 2008 to December 2010 for the 1994–1995 birth cohorts). The HILMO register is virtually complete for all the outpatient and inpatient ICD-10 diagnoses made by Finnish healthcare since 2000 and 1996, respectively.^35^ The cases identified in the register linkage were confirmed by medical history data, which were obtained based on the informed consent from the hospitals and hospital municipal health centers. The most important consideration for not excluding possible causality was temporal association, namely onset within 18 months from the first vaccine dose (i.e., approximately 12 months from the last vaccine dose). For the NOAD assessment, the investigator was blinded to both vaccination and community status.

### Statistical analyses

This was a planned interim analysis, which was initiated after completion of the Month 12 active surveillance, and was conducted when Month 12 passive surveillance data for subjects enrolled by the end of 2009 were available. The current report presents the safety data recorded up to April 2011 in all male and female study participants. The primary population for the analysis of safety was the total vaccinated cohort. For the male diary card subset, the overall percentage of doses with any and grade 3 solicited local and general symptoms were calculated. The percentage of subjects with unsolicited AEs (within 30 d following any vaccine dose) and with SAEs, MSCs and NOADs (up to Month 12 after first vaccine dose) were also calculated. For all study participants (active and passive surveillance combined), the incidence rates (per 100,000 person-years) of subjects with at least one report of SAE with relationship to vaccination over the duration of follow-up for this interim analysis, was calculated with exact 95% CI, together with RR (and 95% CI). The incidence rate (per 100,000 person-years) of study participants with at least one report of NOAD classified by MedDRA was calculated with exact 95% CI, together with RR (and 95% CI), both for the entire duration of follow-up for this interim analysis and for the 12-month period after administration of the last vaccine dose. Relative risks (and 95% CI) as well as 95% CIs around frequency of pregnancy outcomes were calculated in a *post-hoc* analysis.

## Trademark statement

*Cervarix* is a registered trademark of the GSK group of companies. *Engerix-B* is a trademark of the GSK group of companies.

## Highlights

- As seen in girls, the HPV-16/18 vaccine has acceptable safety/reactogenicity in boys
- Similar rates of possibly related serious adverse events for HBV and HPV-16/18 vaccines
- Low incidence of insulin-dependent diabetes mellitus seen in HPV-16/18 vaccinees
- Small number of cases observed for most other new-onset autoimmune diseases
- No apparent differences in incidences of other autoimmune diseases between vaccines

## Supplementary Material

Supplemental_Material.zip

## Abbreviations

AE: adverse event
CI: confidence interval
CIN: cervical intraepithelial neoplasia
HBV: hepatitis B virus
HILMO: Care Register for Social Welfare and Health Care
HPV: human papillomavirus
ICD-10: International Classification of Diseases-10
IDDM: insulin-dependent diabetes mellitus
JA: juvenile arthritis
MPL: 3-*O*-desacyl-4′-monophosphoryl lipid A
MSC: medically significant condition
NOAD: new-onset autoimmune disease
pIMD: potential immune-mediated disease
RR: relative risk
SAE: serious adverse event
VE: vaccine efficacy
VLP: virus-like particle
