# Supplementary material for: Safety of the human papillomavirus (HPV)-16/18 AS04-adjuvanted vaccine in adolescents aged 12–15 years: Interim analysis of a large community-randomized controlled trial
Source: Hum Vaccin Immunother. 2016 Nov 14;12(12):3177–85. doi: 10.1080/21645515.2016.1183847 (PMC5215585; doi:10.1080/21645515.2016.1183847)
Supplement: Supplemental_Material.zip [file khvi-12-12-1183847-s001.zip › HPV-040_HVI SUBMISSION Supplementary Figure 1.pptx]

## Slide 1
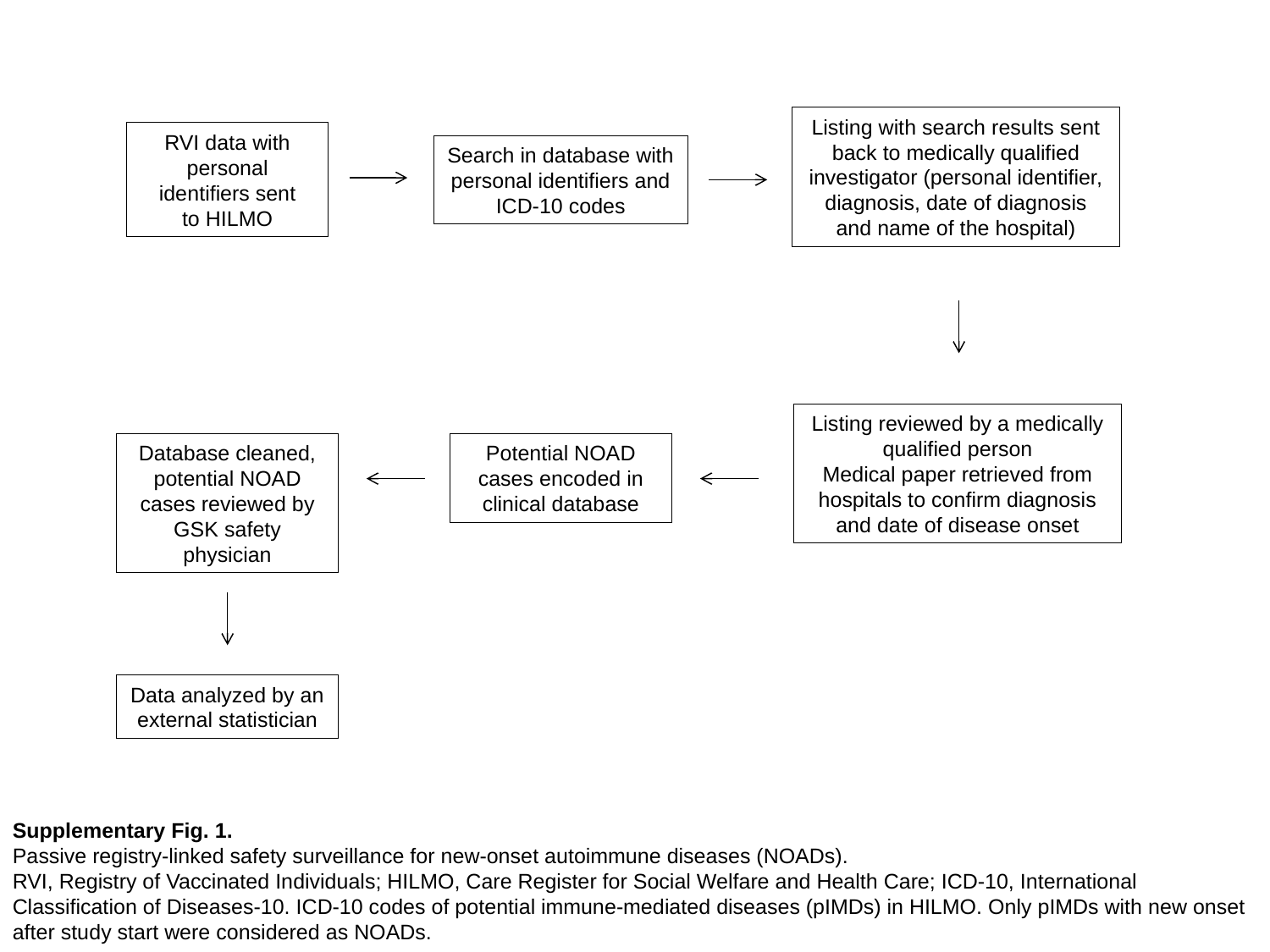

Listing with search results sent back to medically qualified investigator (personal identifier, diagnosis, date of diagnosis and name of the hospital)
RVI data with personal identifiers sent
to HILMO
Search in database with personal identifiers and ICD-10 codes
Listing reviewed by a medically qualified person
Medical paper retrieved from hospitals to confirm diagnosis and date of disease onset
Database cleaned, potential NOAD cases reviewed by GSK safety physician
Potential NOAD cases encoded in clinical database
Data analyzed by an external statistician
Supplementary Fig. 1.
Passive registry-linked safety surveillance for new-onset autoimmune diseases (NOADs).
RVI, Registry of Vaccinated Individuals; HILMO, Care Register for Social Welfare and Health Care; ICD-10, International Classification of Diseases-10. ICD-10 codes of potential immune-mediated diseases (pIMDs) in HILMO. Only pIMDs with new onset after study start were considered as NOADs.
