# Supplementary material for: Safety of the human papillomavirus (HPV)-16/18 AS04-adjuvanted vaccine in adolescents aged 12–15 years: Interim analysis of a large community-randomized controlled trial
Source: Hum Vaccin Immunother. 2016 Nov 14;12(12):3177–85. doi: 10.1080/21645515.2016.1183847 (PMC5215585; doi:10.1080/21645515.2016.1183847)
Supplement: Supplemental_Material.zip [file khvi-12-12-1183847-s001.zip › HPV-040_HVI SUBMISSION Supplementary Table 1.doc]

**Supplementary Table 1.** Summary of baseline demographic characteristics (total vaccinated cohort)

|  | **HPV-16/18 vaccine (N=14,838)** | **HBV  vaccine (N=17,338)** | **Total  (N=32,176)** |
| --- | --- | --- | --- |
| Age at vaccination (dose 1), years  Mean (SD)  Minimum–Maximum | 14.1 (0.75) 12–16 | 14.1 (0.76) 12–16 | 14.1 (0.76) 12–16 |
| Gender, n (%)  Male  Female | 2,440 (16.4) 12,398 (83.6) | 9,221 (53.2) 8117 (46.8) | 11,661 (36.2)  20,515 (63.8) |
| Ethnicity, n (%)  African / African American  Asian (Central / South / East Asian)  Asian (Japanese)  Asian (South East Asian)  White (Arabic / North African)  White (Caucasian / European)  Other | 9 (0.1) 11 (0.1) 1 (0.0) 13 (0.1) 31 (0.2) 14,670 (98.9) 103 (0.7) | 8 (0.0) 4 (0.0) 1 (0.0) 4 (0.0) 39 (0.2) 17,190 (99.1) 92 (0.5) | 17 (0.1) 15 (0.0) 2 (0.0) 17 (0.1) 70 (0.2) 31,860 (99.0) 195 (0.6) |

HPV-16/18 vaccine, HPV-16/18 AS04-adjuvanted vaccine; HBV, hepatitis B virus vaccine; *N,* total number of subjects; *n,* number of subjects in a given category; %, *n/N;* SD, standard deviation.
