# Supplementary material for: Safety of the human papillomavirus (HPV)-16/18 AS04-adjuvanted vaccine in adolescents aged 12–15 years: Interim analysis of a large community-randomized controlled trial
Source: Hum Vaccin Immunother. 2016 Nov 14;12(12):3177–85. doi: 10.1080/21645515.2016.1183847 (PMC5215585; doi:10.1080/21645515.2016.1183847)
Supplement: Supplemental_Material.zip [file khvi-12-12-1183847-s001.zip › HPV-040_HVI SUBMISSION Supplementary Table 2.doc]

**Supplementary Table 2.** Incidence (per 100,000 person years) and relative risk of new-onset insulin-dependent diabetes mellitus (IDDM) considered possibly related to vaccination, stratified according to reporting period and gender.

|  |  |  | **HPV-16/18 vaccine** | | **HBV vaccine** | |  | |
| --- | --- | --- | --- | --- | --- | --- | --- | --- |
|  |  |  | **No. of cases (FU time)** | **Incidence (/100,000)** | **No. of cases (FU time)** | **Incidence (/100,000)** | **Relative risk** | **95% CI** |
| All cases | | Overall | 3 (35991.9) | 8.3 | 21 (41401.2) | 50.7 | 0.16 | 0.03–0.55 |
|  |  | Female | 2 (30318.8) | 6.6 | 7 (19649.6) | 35.6 | 0.19 | 0.02–0.97 |
|  |  | Male | 1 (5673.2) | 17.6 | 14 (21751.6) | 64.4 | 0.27 | 0.01–1.80 |
| Cases occurring within 12 months of vaccination | | Overall | 3 (21421.5) | 14.0 | 8 (25096.5) | 31.9 | 0.44 | 0.08–1.83 |
|  |  | Female | 2 (17950.7) | 11.1 | 5 (11778.4) | 42.5 | 0.26 | 0.02–1.60 |
|  |  | Male | 1 (3470.8) | 28.8 | 3 (13318.1) | 22.5 | 1.28 | 0.02–15.93 |

HPV-16/18 vaccine, HPV-16/18 AS04-adjuvanted vaccine; HBV, hepatitis B virus vaccine; FU*,* follow-up.
