# Supplementary material for: Safety of the human papillomavirus (HPV)-16/18 AS04-adjuvanted vaccine in adolescents aged 12–15 years: Interim analysis of a large community-randomized controlled trial
Source: Hum Vaccin Immunother. 2016 Nov 14;12(12):3177–85. doi: 10.1080/21645515.2016.1183847 (PMC5215585; doi:10.1080/21645515.2016.1183847)
Supplement: Supplemental_Material.zip [file khvi-12-12-1183847-s001.zip › HPV-040_HVI SUBMISSION Supplementary Table 3.doc]

**Supplementary Table 3.** List of the potential immune mediated diseases (pIMD) diagnoses used in the health registry analysis.

| **Neuroinflammatory disorders** | **Musculoskeletal disorders** | | **Skin disorders** |
| --- | --- | --- | --- |
| - Cranial nerve disorders, including paralyses/paresis (e.g. Bell’s palsy), and neuritis (e.g. optic neuritis) - Multiple sclerosis (including variants) - Transverse myelitis - Guillain-Barré syndrome, (including Miller Fisher syndrome and other variants) - Other demyelinating diseases (including acute disseminated encephalomyelitis) - Myasthenia gravis (including Lambert-Eaton myasthenic syndrome) - Non-infectious encephalitis/ encephalomyelitis - Neuritis (including peripheral neuropathies) - Narcolepsy | - Systemic lupus erythematosus - Scleroderma (including, CREST syndrome and morphoea) - Systemic sclerosis - Dermatomyositis - Polymyositis - Antisynthetase syndrome - Rheumatoid arthritis, - Juvenile chronic arthritis, (including Still’s disease) - Polymyalgia rheumatica - Reactive arthritis - Psoriatic arthropathy - Ankylosing spondylitis - Relapsing polychondritis - Mixed connective tissue disorder | | - Psoriasis - Vitiligo - Raynaud’s phenomenon - Erythema nodosum - Autoimmune bullous skin diseases (including pemphigus, pemphigoid and dermatitis herpetiformis) - Cutaneous lupus erythematosus - Alopecia areata - Lichen planus - Sweet’s syndrome |
| **Liver disorders** | **Gastrointestinal disorders** | | **Metabolic diseases** |
| - Autoimmune hepatitis - Primary biliary cirrhosis - Primary sclerosing cholangitis - Autoimmune cholangitis. | - Crohn’s disease - Ulcerative colitis - Ulcerative proctitis - Celiac disease | | - Autoimmune thyroiditis (including Hashimoto thyroiditis) - Grave's or Basedow’s disease - Diabetes mellitus type I - Addison’s disease |
| **Vasculitides** | | **Others** | |
| - Large vessels vasculitis including: giant cell arteritis such as Takayasu's arteritis and temporal arteritis. - Medium sized and/or small vessels vasculitis including: polyarteritis nodosa, Kawasaki's disease, microscopic polyangiitis, Wegener's granulomatosis, Churg–Strauss syndrome, thromboangiitis obliterans (Buerger’s disease), necrotizing vasculitis, allergic granulomatous angiitis, Henoch-Schonlein purpura, anti-neutrophil cytoplasmic antibody positive vasculitis, Behcet's syndrome, leukocytoclastic vasculitis. - Vasculitides secondary to other immune mediated diseases such as lupus vasculitis and rheumatoid vasculitis. | | - Autoimmune hemolytic anemia - Autoimmune thrombocytopenias - Antiphospholipid syndrome - Pernicious anemia - Autoimmune glomerulonephritis (including IgA nephropathy, glomerulonephritis rapidly progressive, membranous glomerulonephritis, membranoproliferative glomerulonephritis, and mesangioproliferative glomerulonephritis) - Uveitis - Autoimmune myocarditis/cardiomyopathy - Sarcoidosis - Stevens-johnson syndrome - Sjögren’s syndrome - Idiopathic pulmonary fibrosis - Goodpasture syndrome | |
